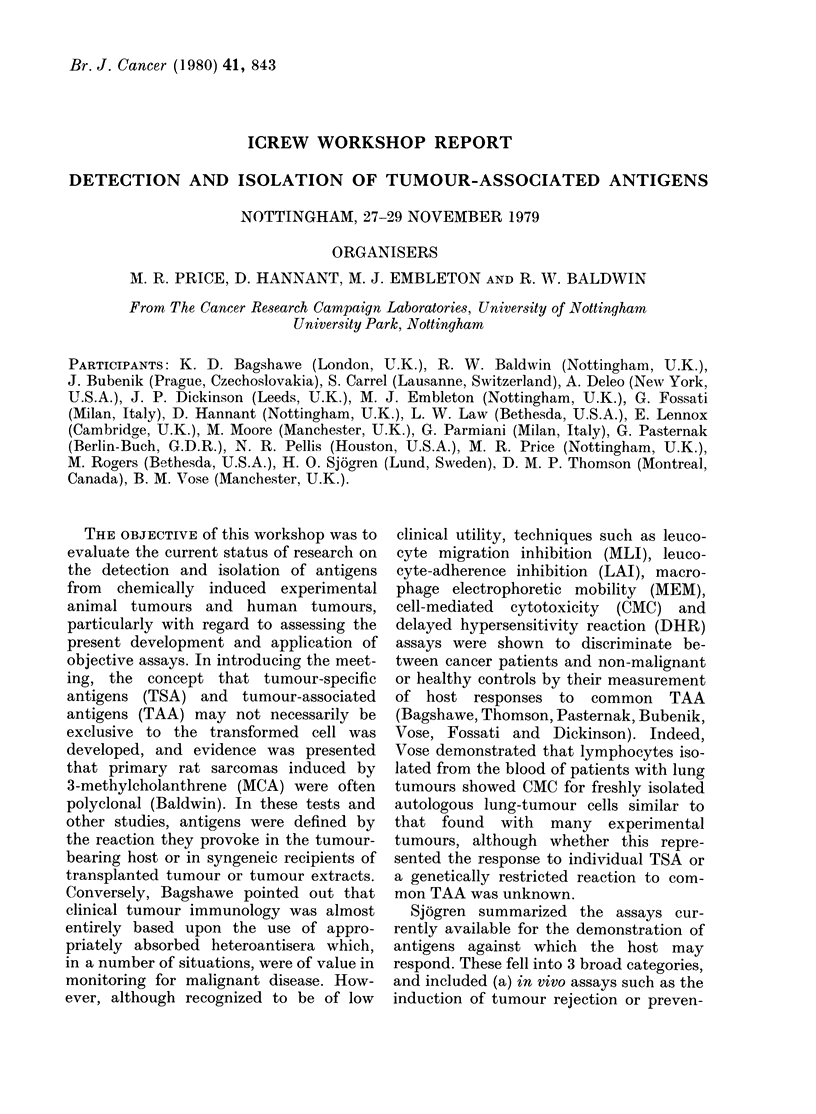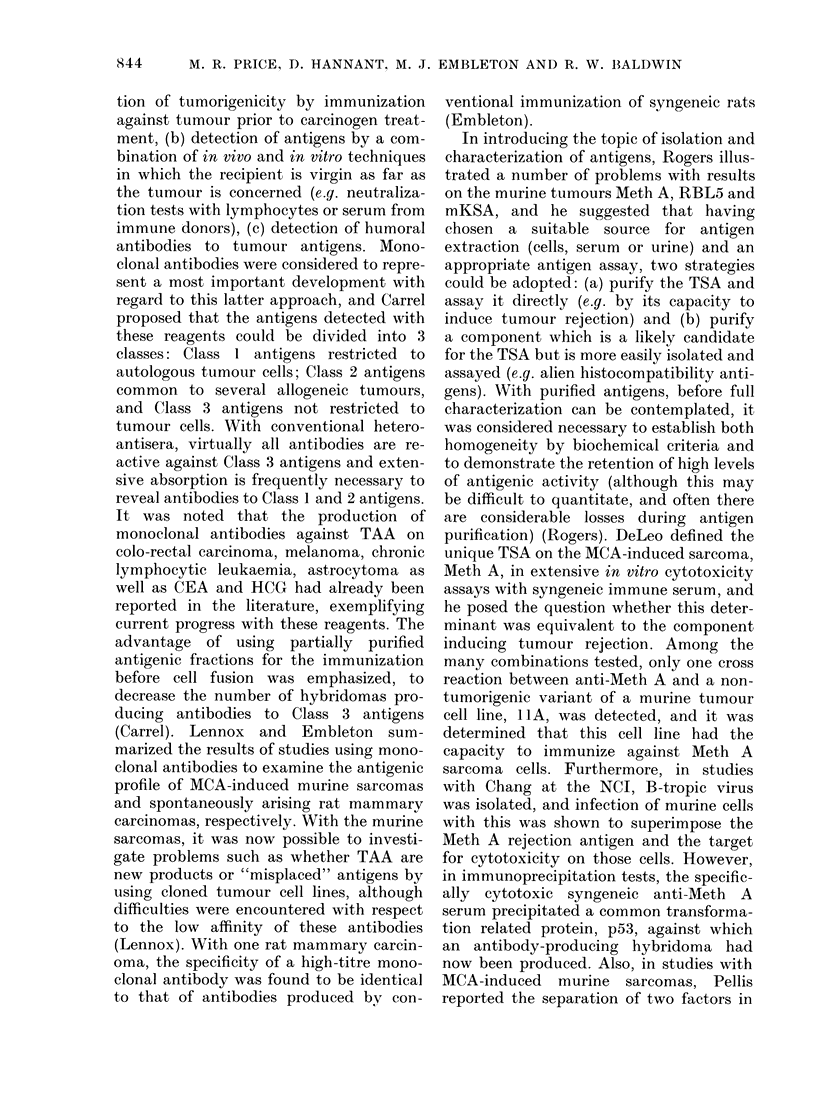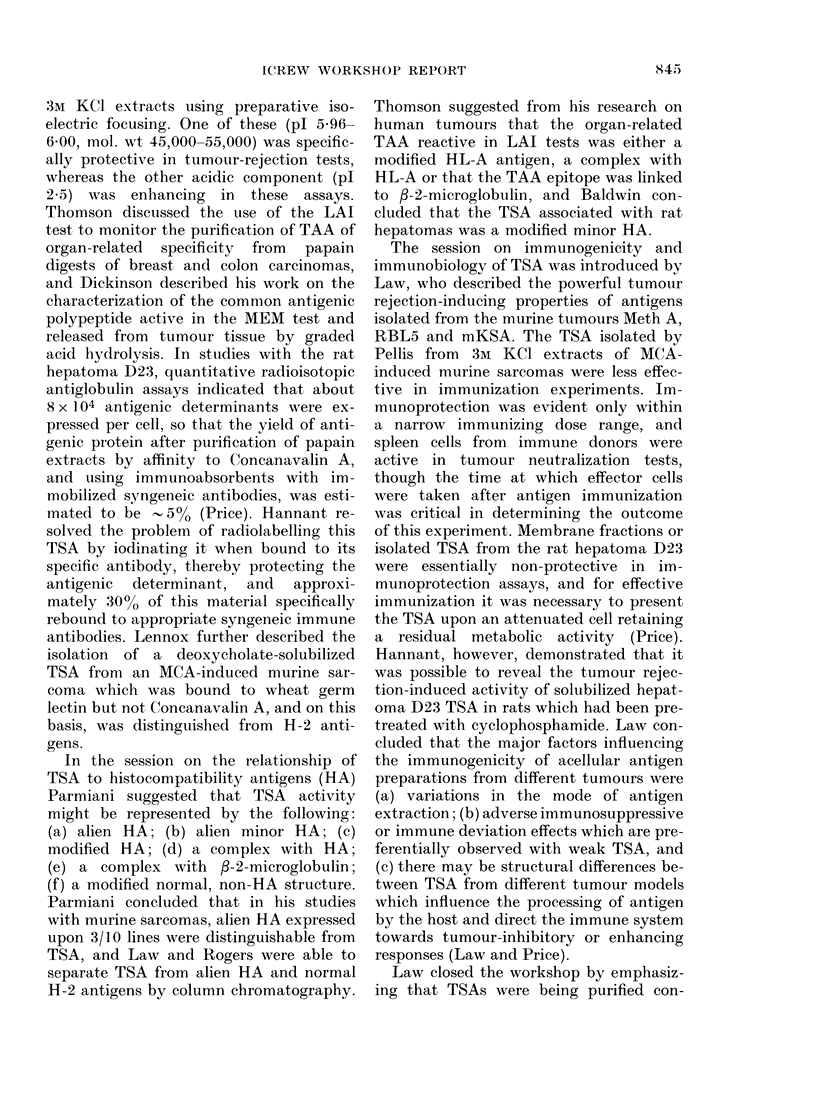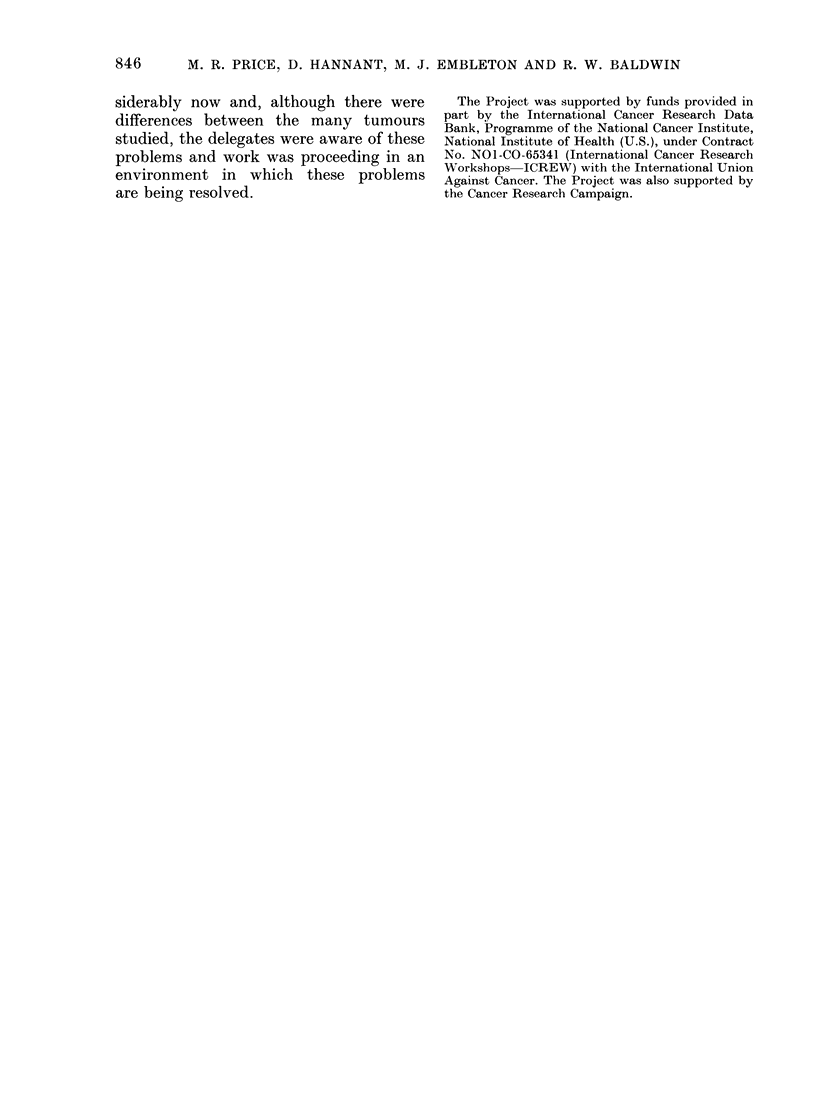# ICREW Workshop Report Detection and isolation of tumour-associated antigens

**Published:** 1980-05

**Authors:** 


*Br. J. Cancer* (1980) **41**, 843

### ICREW WORKSHOP REPORT

### DETECTION AND ISOLATION OF TUMOUR-ASSOCIATED ANTIGENS

NOTTINGHAM, 27–29 NOVEMBER 1979

ORGANISERS

M. R. PRICE, D. HANNANT, M. J. EMBLETON AND R. W. BALDWIN

*From The Cancer Research Campaign Laboratories, University of Nottingham University Park, Nottingham*

PARTICIPANTS: K. D. Bagshawe (London, U.K.), R. W. Baldwin (Nottingham, U.K.), J. Bubenik (Prague, Czechoslovakia), S. Carrel (Lausanne, Switzerland), A. Deleo (New York, U.S.A.), J. P. Dickinson (Leeds, U.K.), M. J. Embleton (Nottingham, U.K.), G. Fossati (Milan, Italy), D. Hannant (Nottingham, U.K.), L. W. Law (Bethesda, U.S.A.), E. Lennox (Cambridge, U.K.), M. Moore (Manchester, U.K.), G. Parmiani (Milan, Italy), G. Pasternak (Berlin-Buch, G.D.R.), N. R. Pellis (Houston, U.S.A.), M. R. Price (Nottingham, U.K.), M. Rogers (Bethesda, U.S.A.), H. O. Sjögren (Lund, Sweden), D. M. P. Thomson (Montreal, Canada), B. M. Vose (Manchester, U.K.).

THE OBJECTIVE of this workshop was to evaluate the current status of research on the detection and isolation of antigens from chemically induced experimental animal tumours and human tumours, particularly with regard to assessing the present development and application of objective assays. In introducing the meeting, the concept that tumour-specific antigens (TSA) and tumour-associated antigens (TAA) may not necessarily be exclusive to the transformed cell was developed, and evidence was presented that primary rat sarcomas induced by 3-methylcholanthrene (MCA) were often polyclonal (Baldwin). In these tests and other studies, antigens were defined by the reaction they provoke in the tumour-bearing host or in syngeneic recipients of transplanted tumour or tumour extracts. Conversely, Bagshawe pointed out that clinical tumour immunology was almost entirely based upon the use of appropriately absorbed heteroantisera which, in a number of situations, were of value in monitoring for malignant disease. However, although recognized to be of low clinical utility, techniques such as leucocyte migration inhibition (MLI), leucocyte-adherence inhibition (LAI), macrophage electrophoretic mobility (MEM), cell-mediated cytotoxicity (CMC) and delayed hypersensitivity reaction (DHR) assays were shown to discriminate between cancer patients and non-malignant or healthy controls by their measurement of host responses to common TAA (Bagshawe, Thomson, Pasternak, Bubenik, Vose, Fossati and Dickinson). Indeed, Vose demonstrated that lymphocytes isolated from the blood of patients with lung tumours showed CMC for freshly isolated autologous lung-tumour cells similar to that found with many experimental tumours, although whether this represented the response to individual TSA or a genetically restricted reaction to common TAA was unknown.

Sjögren summarized the assays currently available for the demonstration of antigens against which the host may respond. These fell into 3 broad categories, and included (a) *in vivo* assays such as the induction of tumour rejection or prevention of tumorigenicity by immunization against tumour prior to carcinogen treatment, (b) detection of antigens by a combination of *in vivo* and *in vitro* techniques in which the recipient is virgin as far as the tumour is concerned (*e.g.* neutralization tests with lymphocytes or serum from immune donors), (c) detection of humoral antibodies to tumour antigens. Monoclonal antibodies were considered to represent a most important development with regard to this latter approach, and Carrel proposed that the antigens detected with these reagents could be divided into 3 classes: Class 1 antigens restricted to autologous tumour cells; Class 2 antigens common to several allogeneic tumours, and Class 3 antigens not restricted to tumour cells. With conventional heteroantisera, virtually all antibodies are reactive against Class 3 antigens and extensive absorption is frequently necessary to reveal antibodies to Class 1 and 2 antigens. It was noted that the production of monoclonal antibodies against TAA on colo-rectal carcinoma, melanoma, chronic lymphocytic leukaemia, astrocytoma as well as CEA and HCG had already been reported in the literature, exemplifying current progress with these reagents. The advantage of using partially purified antigenic fractions for the immunization before cell fusion was emphasized, to decrease the number of hybridomas producing antibodies to Class 3 antigens (Carrel). Lennox and Embleton summarized the results of studies using monoclonal antibodies to examine the antigenic profile of MCA-induced murine sarcomas and spontaneously arising rat mammary carcinomas, respectively. With the murine sarcomas, it was now possible to investigate problems such as whether TAA are new products or "misplaced" antigens by using cloned tumour cell lines, although difficulties were encountered with respect to the low affinity of these antibodies (Lennox). With one rat mammary carcinoma, the specificity of a high-titre monoclonal antibody was found to be identical to that of antibodies produced by conventional immunization of syngeneic rats (Embleton).

In introducing the topic of isolation and characterization of antigens, Rogers illustrated a number of problems with results on the murine tumours Meth A, RBL5 and mKSA, and he suggested that having chosen a suitable source for antigen extraction (cells, serum or urine) and an appropriate antigen assay, two strategies could be adopted: (a) purify the TSA and assay it directly (*e.g.* by its capacity to induce tumour rejection) and (b) purify a component which is a likely candidate for the TSA but is more easily isolated and assayed (*e.g.* alien histocompatibility antigens). With purified antigens, before full characterization can be contemplated, it was considered necessary to establish both homogeneity by biochemical criteria and to demonstrate the retention of high levels of antigenic activity (although this may be difficult to quantitate, and often there are considerable losses during antigen purification) (Rogers). DeLeo defined the unique TSA on the MCA-induced sarcoma, Meth A, in extensive *in vitro* cytotoxicity assays with syngeneic immune serum, and he posed the question whether this determinant was equivalent to the component inducing tumour rejection. Among the many combinations tested, only one cross reaction between anti-Meth A and a non-tumorigenic variant of a murine tumour cell line, 11A, was detected, and it was determined that this cell line had the capacity to immunize against Meth A sarcoma cells. Furthermore, in studies with Chang at the NCI, B-tropic virus was isolated, and infection of murine cells with this was shown to superimpose the Meth A rejection antigen and the target for cytotoxicity on those cells. However, in immunoprecipitation tests, the specifically cytotoxic syngeneic anti-Meth A serum precipitated a common transformation related protein, p53, against which an antibody-producing hybridoma had now been produced. Also, in studies with MCA-induced murine sarcomas, Pellis reported the separation of two factors in

3M KCl extracts using preparative isoelectric focusing. One of these (pI 5·96–6·00, mol. wt 45,000–55,000) was specifically protective in tumour-rejection tests, whereas the other acidic component (pI 2·5) was enhancing in these assays. Thomson discussed the use of the LAI test to monitor the purification of TAA of organ-related specificity from papain digests of breast and colon carcinomas, and Dickinson described his work on the characterization of the common antigenic polypeptide active in the MEM test and released from tumour tissue by graded acid hydrolysis. In studies with the rat hepatoma D23, quantitative radioisotopic antiglobulin assays indicated that about $8 \times 10^4$ antigenic determinants were expressed per cell, so that the yield of antigenic protein after purification of papain extracts by affinity to Concanavalin A, and using immunoabsorbents with immobilized syngeneic antibodies, was estimated to be $\sim 5\%$ (Price). Hannant resolved the problem of radiolabelling this TSA by iodinating it when bound to its specific antibody, thereby protecting the antigenic determinant, and approximately 30% of this material specifically rebound to appropriate syngeneic immune antibodies. Lennox further described the isolation of a deoxycholate-solubilized TSA from an MCA-induced murine sarcoma which was bound to wheat germ lectin but not Concanavalin A, and on this basis, was distinguished from H-2 antigens.

In the session on the relationship of TSA to histocompatibility antigens (HA) Parmiani suggested that TSA activity might be represented by the following: (a) alien HA; (b) alien minor HA; (c) modified HA; (d) a complex with HA; (e) a complex with $\beta$-2-microglobulin; (f) a modified normal, non-HA structure. Parmiani concluded that in his studies with murine sarcomas, alien HA expressed upon 3/10 lines were distinguishable from TSA, and Law and Rogers were able to separate TSA from alien HA and normal H-2 antigens by column chromatography. Thomson suggested from his research on human tumours that the organ-related TAA reactive in LAI tests was either a modified HL-A antigen, a complex with HL-A or that the TAA epitope was linked to $\beta$-2-microglobulin, and Baldwin concluded that the TSA associated with rat hepatomas was a modified minor HA.

The session on immunogenicity and immunobiology of TSA was introduced by Law, who described the powerful tumour rejection-inducing properties of antigens isolated from the murine tumours Meth A, RBL5 and mKSA. The TSA isolated by Pellis from 3M KCl extracts of MCA-induced murine sarcomas were less effective in immunization experiments. Immunoprotection was evident only within a narrow immunizing dose range, and spleen cells from immune donors were active in tumour neutralization tests, though the time at which effector cells were taken after antigen immunization was critical in determining the outcome of this experiment. Membrane fractions or isolated TSA from the rat hepatoma D23 were essentially non-protective in immunoprotection assays, and for effective immunization it was necessary to present the TSA upon an attenuated cell retaining a residual metabolic activity (Price). Hannant, however, demonstrated that it was possible to reveal the tumour rejection-induced activity of solubilized hepatoma D23 TSA in rats which had been pretreated with cyclophosphamide. Law concluded that the major factors influencing the immunogenicity of acellular antigen preparations from different tumours were (a) variations in the mode of antigen extraction; (b) adverse immunosuppressive or immune deviation effects which are preferentially observed with weak TSA, and (c) there may be structural differences between TSA from different tumour models which influence the processing of antigen by the host and direct the immune system towards tumour-inhibitory or enhancing responses (Law and Price).

Law closed the workshop by emphasizing that TSAs were being purified considerably now and, although there were differences between the many tumours studied, the delegates were aware of these problems and work was proceeding in an environment in which these problems are being resolved.

The Project was supported by funds provided in part by the International Cancer Research Data Bank, Programme of the National Cancer Institute, National Institute of Health (U.S.), under Contract No. NO1-CO-65341 (International Cancer Research Workshops—ICREW) with the International Union Against Cancer. The Project was also supported by the Cancer Research Campaign.